# Enhancing voluntary blood donations: King Fahad Armed Forces Hospital experience with mobile donation campaign

**DOI:** 10.3389/fpubh.2026.1762000

**Published:** 2026-02-05

**Authors:** Waleed M. Bawazir, Osama A. Alzahrani, Ahmed G. Bukhari, Raed I. Felimban, Waseem A. Alsolaimi, Naif A. Algarni, Abdulaziz A. Albarqi, Mohammed S. Alzahrani, Nabeel S. Sendi, Junaid H. Faqih, Shaza M. Al-Hindi, Mohammad H. Alhashmi, Hadeel Al Sadoun, Saeed M. Kabrah, Wajnat A. Tounsi, Nora Y. Hakami

**Affiliations:** 1 Department of Medical Laboratory Sciences, Faculty of Applied Medical Sciences, King Abdulaziz University, Jeddah, Saudi Arabia; 2 Hematology Research Unit, King Fahd Medical Research Center, King Abdulaziz University, Jeddah, Saudi Arabia; 3 Blood Bank Laboratory, King Fahad Armed Forces Hospital, Jeddah, Saudi Arabia; 4 3D Bioprinting Unit, Center of Innovation in Personalized Medicine, King Abdulaziz University, Jeddah, Saudi Arabia; 5 Toxicology and Forensic Sciences Unit, King Fahd Medical Research Center, King Abdulaziz University, Jeddah, Saudi Arabia; 6 Nanomedicine Unit, Center of Innovation in Personalized Medicine, King Abdulaziz University, Jeddah, Saudi Arabia; 7 Department of Clinical Laboratory Sciences, Faculty of Applied Medical Sciences, Umm Al-Qura University, Makkah, Saudi Arabia

**Keywords:** blood donation, donor satisfaction, King Fahd Armed Forces Hospital, mobile donation campaign, repeat donors, voluntary donations

## Abstract

**Background:**

Blood donation is crucial for healthcare systems worldwide, ensuring the availability of blood and its products for patients in need. Many regions struggle to maintain adequate blood supplies due to reliance on patient-related donations, leading to shortages and logistical challenges. Increasing voluntary, non-remunerated blood donations (VNRBD) is pivotal for a stable blood supply. Mobile blood donation campaigns have shown promise in enhancing voluntary donation rates by providing convenient opportunities for potential donors. This study aimed to evaluate donor satisfaction levels and compare experiences between first-time and repeat donors in a mobile blood donation campaign conducted at King Fahd Armed Forces Hospital (KFAFH) in Jeddah, Saudi Arabia.

**Materials and methods:**

A cross-sectional study was conducted from October 12, 2023, to February 25, 2024, including 384 adult donors. Data were collected using a structured questionnaire assessing multiple aspects of the donation experience. Descriptive statistics were used to report frequencies, percentages, and mean ± standard deviation (SD) for overall satisfaction. Subgroup analyses compared satisfaction levels between repeat and first-time donors, and between male and female participants.

**Results:**

Donors reported high overall satisfaction, with a mean satisfaction score of 4.5 ± 0.5 (Likert scale 1–5). The vast majority of donors (86.5%) were repeat donors, who reported slightly higher satisfaction (4.6 ± 0.5) compared to first-time donors (4.4 ± 0.6). High satisfaction was observed across all aspects of the donation process, including welcome, clarity of pre-donation survey questions, hygiene, and post-donation care. Both male and female donors reported similarly high satisfaction levels.

**Conclusion:**

The findings highlight the effectiveness of mobile blood donation campaigns in enhancing voluntary blood donations, fostering donor loyalty, and reducing dependency on patient-related donors. The campaign successfully provided a positive experience across donor subgroups, supporting donor retention and engagement.

## 1 Introduction

Blood donation is a critical component of healthcare systems worldwide, ensuring the availability of blood and its components for patients in need. The demand for blood is constant and can only be met through the voluntary efforts of donors. According to the World Health Organization (WHO), safe and adequate blood supplies are essential for the management of trauma, surgical procedures, obstetric emergencies, and chronic conditions such as cancer and hematological disorders (1).

Despite its importance, many regions continue to face challenges in maintaining sufficient blood supplies. One of the main challenges is the reliance on patient-related or replacement donations, in which family members or friends donate blood for a specific patient (2, 3). This practice often results in inequitable access to blood, logistical delays, and increased emotional burden on patients and their families. In contrast, voluntary, non-remunerated blood donation (VNRBD) is widely recognized as the safest and most sustainable source of blood supply (3–5).

From a behavioral perspective, blood donation decisions are influenced by a complex interaction of individual, social, and service-related factors. The Theory of Planned Behavior (TPB) suggests that health-related behaviors are shaped by individuals’ attitudes toward the behavior, perceived social norms, and perceived behavioral control (6). In the context of blood donation, positive attitudes, supportive norms, and perceptions of convenience and safety may enhance donors’ intention to donate and to return for future donations. Similarly, the Health Belief Model (HBM) emphasizes perceived benefits, perceived barriers, and cues to action as key determinants of preventive health behaviors (7). Mobile blood donation campaigns have emerged as an effective strategy to enhance voluntary blood donation by bringing donation services closer to donors’ daily environments, such as workplaces, military bases, and community centers (8). These campaigns not only improve accessibility but also offer opportunities to improve donor experience through streamlined processes and targeted education (9). In Saudi Arabia, the integration of mobile blood donation units with digital platforms—such as the national Wateen application—has further facilitated donor registration, appointment scheduling, and follow-up communication, potentially enhancing donor engagement and satisfaction (10, 11).

In addition to behavioral factors, service-related experiences play a crucial role in shaping donor satisfaction and retention. Service Quality and Satisfaction Theory highlights the importance of service attributes—such as staff interaction, communication, waiting time, hygiene, and post-donation care—in influencing satisfaction and loyalty-related behaviors (12). In blood donation settings, positive service experiences have been shown to increase donor satisfaction and the likelihood of repeat donation, thereby supporting a stable and sustainable blood supply (13, 14).

Globally, efforts to increase voluntary blood donations have met with varying degrees of success. High-income countries have seen more stable blood supplies due to well-established donation programs and public awareness campaigns. In contrast, low- and middle-income countries often struggle with lower rates of voluntary donations and higher dependency on patient-related donors (15, 16).

Research has shown that targeted mobile blood donation campaigns can significantly enhance voluntary blood donation rates. Mobile donation units provide convenient opportunities for potential donors, reaching them in their communities and workplaces, thus reducing the inconvenience that might deter donors from donating at fixed sites (17, 18). Additionally, educational campaigns accompanying mobile units can address common misconceptions about blood donation, increasing donor recruitment and retention (19, 20).

The King Fahd Armed Forces Hospital (KFAFH) in Jeddah, Saudi Arabia, has traditionally faced challenges in maintaining a sufficient blood supply. KFAFH, which serves a large and diverse patient population, has seen high reliance on patient-related donations, creating an urgent need to shift towards a more sustainable model of voluntary donations. The hospital recognized this need and launched a comprehensive mobile donation campaign to increase voluntary blood donors and reduce dependency on patient-related donors.

KFAFH initiated the mobile donation campaigns as a multi-phase project from 2010 to 2022. To reach potential donors directly, this project included deploying mobile donation units to various locations, such as military bases, public areas, and corporate offices. The campaign also integrated digital tools, such as the Wateen application, to facilitate donor registration, appointment scheduling, and setting reminders, thereby enhancing the overall donor experience (21, 22).

During the campaign, extensive efforts were made to educate the public about the importance of blood donation through informational materials and awareness events. The campaigns aimed not only to recruit new donors but also to retain existing ones by fostering a culture of regular donation. The results were promising, showing a marked increase in the number of voluntary donations and a corresponding decrease in patient-related donations.

The COVID-19 pandemic posed unprecedented challenges to blood donation efforts worldwide (23–25). Social distancing measures, lockdowns, and fear of infection significantly reduced donor turnout. However, the flexibility of mobile donation units allowed the hospital to adapt to these challenges by implementing stringent health protocols and reassuring donors that safety measures are in place. The continued operation of mobile units during the pandemic was crucial in maintaining an adequate blood supply.

Despite the growing global interest in mobile blood donation campaigns, empirical evidence from the Middle East remains limited, particularly regarding donor satisfaction as a determinant of voluntary, non-remunerated blood donation (5, 26, 27). Most existing studies focus on donor recruitment rates or operational feasibility, with less attention given to donors’ service experiences and their implications for donor retention and sustainable blood supply systems (28, 29). Furthermore, little is known about how the integration of mobile blood donation services with digital health platforms influences donor engagement and satisfaction (21).

The present study addresses these gaps by providing empirical evidence from a large mobile blood donation campaign conducted at King Fahd Armed Forces Hospital in Jeddah, Saudi Arabia. By systematically assessing donor satisfaction across multiple service-quality domains and documenting the integration of mobile donation units with the Wateen digital platform, this study advances the understanding of donor behavior and service quality in mobile blood donation settings. Accordingly, this study aims to evaluate donor satisfaction levels in a mobile blood donation campaign conducted at King Fahd Armed Forces Hospital in Jeddah, Saudi Arabia.

## 2 Materials and methods

### 2.1 Study design, period, and setting

This study employed a cross-sectional design between October 12, 2023, and February 25, 2024, to evaluate donor satisfaction levels in a mobile blood donation campaign at KFAFH. The campaign aimed to enhance voluntary blood donations and reduce dependency on patient-related blood supplies.

### 2.2 Participants

The study included 384 participants who donated blood during the mobile donation campaign. Participants were selected based on their availability and willingness to provide feedback on their donation experience. Inclusion criteria were adult donors (aged 18 and above) who consented to participate in the survey post-donation. Participants were recruited using a convenience sampling approach among eligible donors who participated in the mobile blood donation campaign during the study period.

### 2.3 Data collection

Data were collected using a structured questionnaire designed to assess various aspects of the donor experience. The questionnaire comprised 15 items focusing on satisfaction levels, including the welcome received, clarity of pre-donation survey questions, time taken to complete the questionnaire, benefit from educational materials, initial triage area, communication of vital signs results, employee responsiveness, waiting time, hygiene and sterilization, instructions about donating blood, care during donation, post-donation instructions, snack provided, efforts to ensure comfort, and the overall survey process. Responses were measured on a five-point Likert scale ranging from “Very Satisfied” to “Very Unsatisfied.” The questionnaire used in this study was specifically developed to assess donor satisfaction levels during the mobile blood donation campaign at KFAFH. The questionnaire was reviewed by a panel of experts in transfusion medicine and public health to assess content validity, clarity, and relevance to donor satisfaction in mobile blood donation settings. Minor wording revisions were made based on their feedback prior to data collection. Internal consistency reliability of the questionnaire was assessed using Cronbach’s alpha, which demonstrated good overall reliability (Cronbach’s alpha = 0.89). An English version of the questionnaire has been uploaded as a Supplementary material.

### 2.4 Sample size calculation

The study’s representative sample size was determined using a specified formula (30). It was calculated as follows: $\text{Sample size}\;(n)=\frac{Z_{1-\alpha /2}^{2}\;P(1-P)}{d^{2}}$ = $\frac{{\left(1.96\right)}^{2}(0.50)\;(1-0.50)}{{\left(0.05\right)}^{2}}=384$ participants.

Where, *Z*_1−*α*/2_ represents the standard normal variate (with a *Z* value of 1.96 for a 95 percent confidence level), *P* denotes the response distribution (50%), and *d* signifies the margin of error (5%).

### 2.5 Data analysis

The collected data were analyzed using IBM® SPSS® software. Descriptive statistics were used to summarize participants’ socio-demographic characteristics and satisfaction levels. Socio-demographic variables included gender, age, and nationality. Satisfaction levels were reported as frequencies and percentages for each questionnaire item, while overall satisfaction and subgroup satisfaction scores were calculated as mean ± standard deviation (SD). Descriptive subgroup analyses were conducted to compare satisfaction between repeat vs. first-time donors and male vs. female donors, providing additional insight into donor experience. No inferential statistical tests were conducted, and the analysis focused on descriptive summaries to evaluate patterns in donor satisfaction.

## 3 Results

The study included 384 participants, with a majority being male (305, 79.4%) and a mean age of 34.98 ± 9.59 years. Participants represented diverse nationalities, with the largest groups being Saudi Arabian (33.1%), Indian (9.9%), British (5.5%), and Pakistani (4.9%).

A significant majority (86.5%) were repeat donors, highlighting prior engagement with blood donation activities and the campaign’s success in fostering donor loyalty (Table 1).

**Table 1:** Socio-demographic characteristics of the study participated blood donors.

Variable	Frequency (*n*)	Percentage (%)
Gender		
Male	305	79.4
Female	79	20.6
Age (mean ± std)	34.98 ± 9.59	
Nationality		
Saudi Arabian	127	33.1
Indian	38	9.9
British	21	5.5
Pakistani	19	4.9
Mexican	17	4.4
Filipino	12	3.1
Chinese	12	3.1
Spanish	9	2.3
Portuguese	9	2.3
Greek	8	2.1
Brazilian	8	2.1
Indonesian	8	2.1
British (United Kingdom)	8	2.1
Tunisian	8	2.1
Syrian	8	2.1
United Kingdom	5	1.3
Dutch	5	1.3
Nepali	5	1.3
Austrian	5	1.3
Sri Lankan	4	1.0
Malaysian	4	1.0
Vietnamese	4	1.0
South African	4	1.0
Portuguese	4	1.0
Russian	4	1.0
Korean	4	1.0
French	4	1.0
Slovak	4	1.0
Colombia	4	1.0
Irish	4	1.0
Colombian	4	1.0
Previously donated blood		
Yes	332	86.5
No	52	13.5

Overall, donor satisfaction was high, with an average of 83.9% of participants reporting being very satisfied across all aspects of the donation process (Table 2). Key areas of very high satisfaction included care during donation (92.4%), staff responsiveness (90.1%), and clarity of pre-donation instructions (88.8%). Minor areas for improvement included the post-donation snack provision, with 74.2% of donors very satisfied.

**Table 2:** Blood donor’s satisfaction levels in a mobile blood donation campaign.

Variable	Very satisfied *n* (%)	Satisfied *n* (%)	Neutral *n* (%)	Unsatisfied *n* (%)	Very unsatisfied *n* (%)
The welcome received					
	341 (88.8)	18 (4.7)	9 (2.3)	16 (4.3)	0 (0.0)
The clarity of pre-donation blood survey questions					
	319 (83.1)	42 (10.9)	20 (5.2)	3 (0.8)	0 (0.0)
The time taken to complete the blood donation questionnaire					
	303 (78.9)	71 (18.5)	0 (0.0)	10 (2.6)	0 (0.0)
The beneficiary of educational and awareness publications provided					
	302 (78.6)	46 (12.0)	33 (8.6)	3 (0.8)	0 (0.0)
The initial triage area					
	311 (81.0)	48 (12.5)	25 (6.5)	0 (0.0)	0 (0.0)
The communication of the vital signs results					
	323 (84.1)	51 (13.3)	10 (2.6)	0 (0.0)	0 (0.0)
The employee’s responses to inquiries					
	346 (90.1)	37 (9.6)	1 (0.3)	0 (0.0)	0 (0.0)
The waiting time before being called into the blood collection room					
	324 (84.4)	51 (13.3)	3 (0.8)	6 (1.6)	0 (0.0)
The general hygiene and sterilization maintained					
	331 (86.2)	42 (10.9)	5 (1.3)	6 (1.3)	0 (0.0)
The instructions given about donating blood					
	341 (88.8)	40 (10.4)	1 (0.3)	2 (0.5)	0 (0.0)
The care provided during the blood donation process					
	355 (92.4)	29 (7.6)	0 (0.0)	0 (0.0)	0 (0.0)
The post-donation instructions given					
	333 (86.7)	42 (10.9)	9 (2.3)	0 (0.0)	0 (0.0)
The snack provided after the blood donation					
	285 (74.2)	62 (16.1)	25 (6.5)	12 (3.1)	0 (0.0)
The efforts made to ensure comfort after donating blood					
	312 (81.3)	48 (12.5)	20 (5.2)	4 (1.0)	0 (0.0)
The survey process conducted					
	300 (78.1)	61 (15.9)	20 (5.2)	3 (0.8)	0 (0.0)
Overall (average)	322 (83.9)	46 (12.0)	12 (3.1)	4 (1.0)	0 (0.0)

Descriptive subgroup analysis revealed that repeat donors reported slightly higher satisfaction (Mean ± SD: 4.6 ± 0.5) compared to first-time donors (4.4 ± 0.6), suggesting that prior positive donation experiences may enhance donor satisfaction and reinforce loyalty. Male donors had a marginally higher mean satisfaction score (4.5 ± 0.5) than female donors (4.4 ± 0.6), although both genders reported high overall satisfaction. These findings indicate that the mobile blood donation campaign effectively provided a positive experience across all donor subgroups, supporting donor retention and engagement (Table 3).

**Table 3:** Donor satisfaction levels by subgroup in the mobile blood donation campaign.

Subgroup	Mean satisfaction ± SD	*N* (%) of total
Repeat donors	4.6 ± 0.5	332 (86.5%)
First-time donors	4.4 ± 0.6	52 (13.5%)
Male donors	4.5 ± 0.5	305 (79.4%)
Female donors	4.4 ± 0.6	79 (20.6%)

## 4 Discussion

The findings of this study demonstrate high levels of donor satisfaction with the mobile blood donation campaign conducted at King Fahd Armed Forces Hospital, underscoring the effectiveness of mobile donation services in promoting voluntary blood donation. The exceptionally high satisfaction levels observed in this study may be attributed to several interrelated factors. First, the mobile blood donation setting reduced common logistical barriers such as travel distance, waiting time, and scheduling constraints, which are frequently cited deterrents to blood donation. Second, the high standards of organization, cleanliness, and staff professionalism likely enhanced donors’ perceptions of safety and trust. In healthcare service contexts, particularly those involving invasive procedures such as blood donation, perceived competence and respectful communication by staff play a central role in shaping overall satisfaction. These findings reinforce the importance of donor-centered service delivery in sustaining voluntary blood donation systems.

From a behavioral perspective, these findings align with established donor behavior theories (6, 31, 32). According to the Theory of Planned Behavior, positive service experiences contribute to favorable attitudes toward donation and strengthen perceived behavioral control by minimizing physical and psychological obstacles. Similarly, the Health Belief Model suggests that mobile donation campaigns reduce perceived barriers while enhancing perceived benefits, thereby increasing donors’ willingness to participate. The high proportion of repeat donors in this study supports the notion that satisfaction reinforces donation intentions and long-term donor retention.

The findings are also consistent with the Health Belief Model (7, 33, 34). Mobile donation campaigns may reduce perceived barriers such as travel distance and time constraints, while increasing perceived benefits by offering a convenient, safe, and well-organized donation experience. Educational and awareness materials provided during the campaign likely served as cues to action, reinforcing donors’ motivation to participate in blood donation activities.

The cultural and institutional context of Saudi Arabia may further help explain the high satisfaction and participation levels. Blood donation campaigns in the Kingdom are often framed within broader values of social solidarity, altruism, and religious encouragement to save lives. In addition, the involvement of a well-established military healthcare institution may enhance public trust and credibility, which are critical determinants of donor confidence. The integration of digital tools such as the Wateen application also reflects national efforts to modernize healthcare services and improve donor engagement, contributing to a more seamless and reassuring donation experience.

High donor satisfaction is particularly important in the context of sustaining a stable blood supply, as satisfied donors are more likely to return for future donations and to recommend donation to others. The findings of this study suggest that mobile blood donation campaigns, when supported by high service quality and institutional trust, can serve as an effective strategy for strengthening voluntary blood donation systems.

Service Quality and Satisfaction Theory further provides a useful lens for interpreting the results (35–37). The exceptionally high satisfaction levels reported for staff behavior, hygiene, communication, and care during donation suggest that service quality plays a pivotal role in shaping donor satisfaction and loyalty. Previous research has shown that satisfied donors are more likely to return for future donations, contributing to a stable and reliable blood supply. The high percentage of donors with prior donation experience in this study supports this relationship between satisfaction and donor retention.

The integration of digital tools, particularly the Wateen application, represents an additional strength of the mobile donation campaign. Digital platforms can enhance donor engagement by simplifying registration, scheduling, and follow-up communication, thereby reinforcing positive donor experiences (29, 38, 39). Although this study did not directly measure the independent effect of the digital platform, its integration within the mobile donation process likely contributed to overall satisfaction and operational efficiency.

The use of mobile donation units has been shown to be effective in reaching potential donors and making the donation process more convenient. This approach aligns with findings from studies in other regions, which have demonstrated that mobile units can increase donation rates by bringing the donation process closer to donors’ everyday environments, such as workplaces and community centers (40, 41). By reducing the inconvenience of traveling to fixed donation sites, mobile units can help overcome a significant barrier to donation.

Moreover, integrating digital tools like the Wateen application to streamline donor registration and appointment scheduling was another critical factor in the campaign’s success. This technological integration is supported by other studies that highlight the positive impact of digital solutions on donor engagement and satisfaction (42, 43). By providing an easy and accessible way for donors to manage their donations, the Wateen application likely played a crucial role in enhancing the overall donor experience.

The study also observed a significant reduction in the reliance on patient-related donations, shifting towards a more sustainable model of voluntary donations. This transition is vital for maintaining a stable blood supply and reducing the emotional and logistical burdens on patients and their families. Similar outcomes have been reported in other settings where efforts to promote voluntary, non-remunerated blood donations have led to more reliable blood supplies (5).

The high proportion of repeat donors (86.5%) observed in this study is particularly noteworthy. Repeat donors play a central role in maintaining a reliable blood supply, as they tend to have lower deferral rates, higher compliance with eligibility criteria, and greater trust in donation services. Positive experiences during the mobile donation campaign, including staff responsiveness, efficient organization, and hygienic procedures, likely reinforced donor loyalty. Descriptive subgroup analyses suggested that repeat donors reported slightly higher satisfaction than first-time donors, highlighting the importance of prior positive experiences in encouraging continued donation. These findings emphasize the role of donor satisfaction in promoting retention and sustaining voluntary blood donation programs. Repeat donors are essential for maintaining a consistent blood supply, as they are more likely to donate regularly and are familiar with the donation process (40). This finding aligns with research indicating that positive donor experiences and effective donor recruitment strategies can foster long-term donor loyalty (44).

Despite the success of the mobile donation campaign, challenges remain. The study period coincided with the ongoing COVID-19 pandemic, which posed additional difficulties for blood donation efforts globally. Social distancing measures, lockdowns, and fear of infection significantly impacted donor turnout. However, the flexibility of mobile units allowed the hospital to adapt to these challenges by implementing stringent health protocols and reassuring donors about the safety measures in place. This adaptability was crucial in maintaining an adequate blood supply during the pandemic, as noted in other regions’ studies (45).

Despite the promising findings of this study, several limitations must be acknowledged. First, the study’s cross-sectional design limits the ability to establish causality between the mobile donation campaign and increased donor satisfaction. Longitudinal studies would be beneficial to assess the long-term impact of mobile units on donor retention and blood supply stability.

Second, the study relied on self-reported donor data, which may be subject to response bias. Participants might have provided socially desirable responses, especially regarding their satisfaction with the donation process. Future studies could incorporate more objective measures of satisfaction and engagement to complement self-reported data (46).

Although the sample size was determined using a standard formula, it may not fully capture the diversity of the donor population. While the study included donors from various nationalities, the majority were male, which may not reflect the broader demographic composition of potential donors. This gender imbalance could influence the generalizability of the findings. Recruiting a more balanced sample regarding gender and other demographic factors would provide a more comprehensive understanding of donor experiences and satisfaction (47).

Additionally, the use of convenience sampling within a single healthcare institution may introduce selection bias, as donors who agreed to participate may differ systematically from those who did not. This may limit the generalizability of the findings to the broader donor population. Different hospitals and regions may face unique challenges and have varying levels of resources and infrastructure for implementing mobile donation campaigns. Comparative studies across multiple centers could provide insights into the effectiveness of such campaigns in diverse contexts (48).

Lastly, while the study highlights the benefits of mobile donation units, it does not explore potential drawbacks or logistical challenges associated with their deployment. Issues such as the cost of maintaining mobile units, ensuring staff availability, and addressing potential donor fatigue were not examined. A more detailed cost–benefit analysis could help healthcare institutions make informed decisions about investing in mobile donation infrastructure (49).

### 4.1 Conclusions and recommendations

The mobile blood donation campaign conducted by KFAFH has demonstrated significant success in enhancing voluntary blood donations and reducing reliance on patient-related donors. The campaign’s strategic approach, which included deploying mobile donation units to convenient locations and leveraging digital tools for donor engagement, effectively addressed logistical barriers and increased donor participation. High levels of donor satisfaction were observed across various aspects of the donation process, reflecting the campaign’s ability to provide donors with a positive and comfortable experience. This satisfaction is crucial not only for retaining existing donors but also for encouraging new donors to participate in future donation drives.

Based on our experience, we recommend the following strategies to further improve voluntary blood donation practices:

1. Expand mobile donation coverage by targeting workplaces, universities, and community centers to reach new donor populations.
2. Integrate digital tools such as mobile apps for donor registration, appointment scheduling, reminders, and follow-up communication to enhance convenience and engagement.
3. Strengthen educational campaigns during mobile drives to address misconceptions, increase awareness of the benefits of donation, and provide clear guidance on eligibility criteria.
4. Prioritize donor comfort and service quality, including staff responsiveness, hygiene standards, and post-donation care, as these factors directly influence donor satisfaction and repeat donation.
5. Monitor and evaluate donor feedback continuously to identify areas for improvement and tailor campaigns to different donor subgroups, including first-time and female donors.
6. Foster a culture of repeat donation through recognition programs, loyalty initiatives, and community engagement to build a sustainable pool of voluntary donors.

The experience of KFAFH can serve as a model for other healthcare institutions in Saudi Arabia and the wider region aiming to enhance their blood donation programs. Continued efforts to promote voluntary, non-remunerated blood donations are essential for ensuring a stable and sufficient blood supply. Educational initiatives, community engagement, and innovative technologies will play a critical role in sustaining these efforts and meeting the ongoing demand for blood and its components.

## Data Availability

The raw data supporting the conclusions of this article will be made available by the authors, without undue reservation.

## References

[1] WHO. Global status report on blood safety and availability 2021 World Health Organization (2022).

[2] Laermans J O D den Van Bosch E De Buck E Compernolle V Shinar E . Impact of disasters on blood donation rates and blood safety: a systematic review and meta-analysis. Vox Sang. (2022) 117:769–79. doi: 10.1111/vox.13255, 35167126 PMC9306627

[3] Emuron T. L. Improving the fill rate for blood donor ambassador volunteers at the American Red Cross in Douglas County, NE Nashville, Tennessee, USA: Vanderbilt University (2022).

[4] Fong I Fong I. Blood transfusion-associated infections in the twenty-first century: new challenges In: Current trends and concerns in infectious diseases Ed. Fong IW. Cham, Switzerland: Springer International Publishing / Springer Nature Switzerland AG. (2020). 191–215.

[5] WHO, Towards 100% voluntary blood donation: a global framework for action. Geneva, Switzerland: World Health Organization (2010). Available online at: https://www.who.int/publications/i/item/9789241599696 (Accessed November 12, 2025) 26225399

[6] Ajzen I. The theory of planned behavior. Organ Behav Hum Decis Process. (1991) 50:179–211. doi: 10.1016/0749-5978(91)90020-T

[7] Rosenstock IM. The health belief model and preventive health behavior. Health Educ Monogr. (1974) 2:354–86. doi: 10.1177/109019817400200405

[8] Li L Valero M Keyser R Ukuku AM Zheng D. Mobile applications for encouraging blood donation: a systematic review and case study. Digit Health. (2023) 9:20552076231203603. doi: 10.1177/20552076231203603, 37822963 PMC10563464

[9] Ravindran P. Paramasivam G. Susilo Y. K. B., BloodGo: transforming blood donation services through smart mobile technology. Rochester, New York, USA: Social Science Research Network (SSRN). (Available at SSRN 5416194) (2025).

[10] Benchikh Tasnime BM. Digital health applications and their role in improving the quality of health care services study the experience of Saudi Arabia Mohamed El Bachir El Ibrahimi University of Bordj Bou Arreridj – Faculty of Economic and Commercial Sciences (2024).

[11] Batis AA Albarrak A. Preferences and features of a blood donation smartphone app: a multicenter mixed-methods study in Riyadh, Saudi Arabia. Comput Methods Programs Biomed Update. (2021) 1:100005. doi: 10.1016/j.cmpbup.2021.100005

[12] Parasuraman A Berry LL Zeithaml VA. Refinement and reassessment of the SERVQUAL scale. J Retail. (1991) 67:420. Available online at: https://www.researchgate.net/publication/304344168_Refinement_and_reassessment_of_the_SERVQUAL_scale#fullTextFileContent

[13] Bove LL Bednall T Masser B Buzza M. Blood donors and blood collection. Transfusion. (2011) 51:2411–24. doi: 10.1111/j.1537-2995.2011.03168.x 21564104

[14] France JL France CR Himawan LK. A path analysis of intention to redonate among experienced blood donors: an extension of the theory of planned behavior. Transfusion. (2007) 47:1006–13. doi: 10.1111/j.1537-2995.2007.01236.x 17524090

[15] Nwogoh B Aigberadion U Nwannadi AI. Knowledge, attitude, and practice of voluntary blood donation among healthcare workers at the University of Benin Teaching Hospital, Benin City, Nigeria. J Blood Transfus. (2013) 2013:797830. doi: 10.1155/2013/797830 24222890 PMC3810036

[16] Yin YH Li CQ Liu Z. Blood donation in China: sustaining efforts and challenges in achieving safety and availability. Transfusion. (2015) 55:2523–30. doi: 10.1111/trf.13130, 26111254

[17] Paridar M Khosravi A Jalali-Far M-A Zolfaghari S Ghaleh Sardi OK Sajadi M. Mobile blood collection sites and their roles in providing safe and adequate supply: a six-year experience. Front Biol. (2018) 13:226–34. doi: 10.1007/s11515-018-1497-z

[18] Karadağ İ Keskin ME Yiğit V. Re-design of a blood supply chain organization with mobile units. Soft Comput. (2021) 25:6311–27. doi: 10.1007/s00500-021-05618-3, 33619428 PMC7890397

[19] Guglielmetti Mugion R. Pasca M. G. Pietro L. Renzi M. F. Promoting the propensity for blood donation through the understanding of its determinants BMC Health Serv Res 21 2021 1–20 doi: 10.1186/s12913-021-06134-8 33550982 PMC7868170

[20] Zucoloto ML Gonçalez TT Gilchrist PT Custer B McFarland W Martinez EZ. Factors that contribute to blood donation behavior among primary healthcare users: a structural approach. Transfus Apher Sci. (2019) 58:663–8. doi: 10.1016/j.transci.2019.08.020, 31519527

[21] Alessa T. Evaluation of the Wateen app in the blood-donation process in Saudi Arabia. J Blood Med. (2022) 13:181–90. doi: 10.2147/jbm.s360091, 35450013 PMC9017702

[22] Mohamoud AM. An evaluation of blood transfusion Services of Saudi Arabia. Bristol: Faculty of Applied Sciences, University of the West of England (2024).

[23] Amoguis K. K. S. Booth M. E. A. T. Caballero D. B. Cruz K. E. A. D. Luis M. A. O. Perez A. V. S. ., COVID-19 challenges and associated implemented measures of blood banks: a systematic review. 2022). Quezon City, Metro Manila, Philippines: Far Eastern University – Dr. Nicanor Reyes Medical Foundation.

[24] Dhiman Y Patidar GK Arora S. COVID-19 pandemic-response to challenges by blood transfusion services in India: a review report. ISBT Sci Ser. (2020) 15:365–73. doi: 10.1111/voxs.12563

[25] Al Mahmasani L Hodroj MH Finianos A Taher A. COVID-19 pandemic and transfusion medicine: the worldwide challenge and its implications. Ann Hematol. (2021) 100:1115–22. doi: 10.1007/s00277-021-04441-y, 33527161 PMC7850517

[26] Glynn SA Kleinman SH Schreiber GB Zuck T Mc Combs S Bethel J . Motivations to donate blood: demographic comparisons. Transfusion. (2002) 42:216–25. doi: 10.1046/j.1537-2995.2002.00008.x 11896338

[27] Bednall TC Bove LL Cheetham A Murray AL. A systematic review and meta-analysis of antecedents of blood donation behavior and intentions. Soc Sci Med. (2013) 96:86–94. doi: 10.1016/j.socscimed.2013.07.022, 24034955

[28] Godin G Conner M Sheeran P Bélanger-Gravel A Germain M. Determinants of repeated blood donation among new and experienced blood donors. Transfusion. (2007) 47:1607–15. doi: 10.1111/j.1537-2995.2007.01331.x, 17725724

[29] WHO. Blood donor selection: guidelines on assessing donor suitability for blood donation Hoboken, New Jersey, USA: World Health Organization (2012). 23700651

[30] Charan J Biswas T. How to calculate sample size for different study designs in medical research? Indian J Psychol Med. (2013) 35:121–6. doi: 10.4103/0253-7176.116232, 24049221 PMC3775042

[31] Godin G Sheeran P Conner M Germain M Blondeau D Gagné C . Factors explaining the intention to give blood among the general population. Vox Sang. (2005) 89:140–9. doi: 10.1111/j.1423-0410.2005.00674.x, 16146506

[32] Kowalsky JM France CR France JL Whitehouse EA Himawan LK. Blood donation fears inventory: development and validation of a measure of fear specific to the blood donation setting. Transfus Apher Sci. (2014) 51:146–51. doi: 10.1016/j.transci.2014.07.007, 25151096

[33] Rosenstock IM Strecher VJ Becker MH. Social learning theory and the health belief model. Health Educ Q. (1988) 15:175–83. doi: 10.1177/109019818801500203, 3378902

[34] Lemmens KP Abraham C Hoekstra T Ruiter RA De Kort WL Brug J . Why don’t young people volunteer to give blood? An investigation of the correlates of donation intentions among young nondonors. Transfusion. (2005) 45:945–55. doi: 10.1111/j.1537-2995.2005.04379.x, 15934993

[35] Parasuraman A Zeithaml VA Berry L. SERVQUAL: a multiple-item scale for measuring consumer perceptions of service quality (1988) 64:12–40. Available online at: https://www.researchgate.net/publication/200827786_SERVQUAL_A_Multiple-item_Scale_for_Measuring_Consumer_Perceptions_of_Service_Quality#fullTextFileContent

[36] Bednall TC Bove LL. Donating blood: a meta-analytic review of self-reported motivators and deterrents. Transfus Med Rev. (2011) 25:317–34. doi: 10.1016/j.tmrv.2011.04.005, 21641767

[37] Schreiber GB Sharma U Wright D Glynn S Ownby H Tu Y . First year donation patterns predict long-term commitment for first-time donors. Vox Sang. (2005) 88:114–21. doi: 10.1111/j.1423-0410.2005.00593.x, 15720609

[38] Melián-Alzola L Martín-Santana JD. Service quality in blood donation: satisfaction, trust and loyalty. Serv Bus. (2020) 14:101–29. doi: 10.1007/s11628-019-00411-7

[39] Ferreira A Santos C. Digital transformation in public sector: systematic literature review In: Enhancing public sector accountability and services through digital innovation (2025). Ed. Sang M.L, Berlin and Heidelberg, Germany: Springer Science+Business Media. 265–88.

[40] Glynn SA Williams AE Nass CC Bethel J Kessler D Scott EP . Attitudes toward blood donation incentives in the United States: implications for donor recruitment. Transfusion. (2003) 43:7–16. doi: 10.1046/j.1537-2995.2003.00252.x 12519425

[41] Schlumpf KS Glynn SA Schreiber GB Wright DJ Steele WR Tu Y . Factors influencing donor return. Transfusion. (2008) 48:264–72. doi: 10.1111/j.1537-2995.2007.01519.x, 18005325

[42] Otieno L. Blood donor recruitment and education for successful donor retention: a literature review (2018). Hoboken, New Jersey, USA. doi: 10.58506/ajstss.v2i2.206

[43] Kiarie N Kirongo AC Mwadulo M. A systematic review of predictive blood donor retention models. Afr J Sci Technol Soc Sci. (2023) 2:35–41.

[44] France CR France JL Wissel ME Kowalsky JM Bolinger EM Huckins JL. Enhancing blood donation intentions using multimedia donor education materials. Transfusion. (2011) 51:1796–801. doi: 10.1111/j.1537-2995.2010.03033.x, 21303366

[45] Pagano MB Hess JR Tsang HC Staley E Gernsheimer T Sen N . Prepare to adapt: blood supply and transfusion support during the first 2 weeks of the 2019 novel coronavirus (COVID-19) pandemic affecting Washington state. Transfusion. (2020) 60:908–11. doi: 10.1111/trf.15789 32198754

[46] Bryman A. Social research methods Hoboken, New Jersey, USA: Oxford university press (2016).

[47] Creswell JW Creswell JD. Research design: qualitative, quantitative, and mixed methods approaches Oxford, United Kingdom: Sage publications (2017).

[48] Polit DF Beck CT. Nursing research: generating and assessing evidence for nursing practice Thousand Oaks, California: Lippincott Williams & Wilkins (2008).

[49] Piersma TW Bekkers R Klinkenberg EF De Kort WL Merz E-M. Individual, contextual and network characteristics of blood donors and non-donors: a systematic review of recent literature. Blood Transfus. (2017) 15:382. doi: 10.2450/2017.0064-17 28686151 PMC5589701

