# Supplementary material for: Enhancing voluntary blood donations: King Fahad Armed Forces Hospital experience with mobile donation campaign
Source: Front Public Health. 2026 Feb 5;14:1762000. doi: 10.3389/fpubh.2026.1762000 (PMC12916555; doi:10.3389/fpubh.2026.1762000)
Supplement: Supplementary file 1 [file Table_1.DOCX]

1. **Socio-demographic characteristics**

**Gender: ⃝** Male ⃝ Female

**Age** (Years): **Nationality:
Previously donated blood: ⃝** Yes ⃝ No

1. **Blood donor’s satisfaction levels in a mobile blood donation campaign**

**The welcome received:**

⃝ Very satisfied ⃝ Satisfied ⃝ Neutral ⃝ Unsatisfied ⃝ Very unsatisfied

**The clarity of pre-donation blood survey questions**

⃝ Very satisfied ⃝ Satisfied ⃝ Neutral ⃝ Unsatisfied ⃝ Very unsatisfied

**The time taken to complete the blood donation questionnaire**

⃝ Very satisfied ⃝ Satisfied ⃝ Neutral ⃝ Unsatisfied ⃝ Very unsatisfied

**The beneficiary of educational and awareness publications provided**

⃝ Very satisfied ⃝ Satisfied ⃝ Neutral ⃝ Unsatisfied ⃝ Very unsatisfied

**The initial triage area**

⃝ Very satisfied ⃝ Satisfied ⃝ Neutral ⃝ Unsatisfied ⃝ Very unsatisfied

**The communication of the vital signs results**

⃝ Very satisfied ⃝ Satisfied ⃝ Neutral ⃝ Unsatisfied ⃝ Very unsatisfied

**The employee's responses to inquiries**

⃝ Very satisfied ⃝ Satisfied ⃝ Neutral ⃝ Unsatisfied ⃝ Very unsatisfied

**The waiting time before being called into the blood collection room**

⃝ Very satisfied ⃝ Satisfied ⃝ Neutral ⃝ Unsatisfied ⃝ Very unsatisfied

**The general hygiene and sterilization maintained**

⃝ Very satisfied ⃝ Satisfied ⃝ Neutral ⃝ Unsatisfied ⃝ Very unsatisfied

**The instructions given about donating blood**

⃝ Very satisfied ⃝ Satisfied ⃝ Neutral ⃝ Unsatisfied ⃝ Very unsatisfied

**The care provided during the blood donation process**

⃝ Very satisfied ⃝ Satisfied ⃝ Neutral ⃝ Unsatisfied ⃝ Very unsatisfied

**The post-donation instructions given**

⃝ Very satisfied ⃝ Satisfied ⃝ Neutral ⃝ Unsatisfied ⃝ Very unsatisfied

**The snack provided after the blood donation**

⃝ Very satisfied ⃝ Satisfied ⃝ Neutral ⃝ Unsatisfied ⃝ Very unsatisfied

**The efforts made to ensure comfort after donating blood**

⃝ Very satisfied ⃝ Satisfied ⃝ Neutral ⃝ Unsatisfied ⃝ Very unsatisfied

**The survey process conducted**

⃝ Very satisfied ⃝ Satisfied ⃝ Neutral ⃝ Unsatisfied ⃝ Very unsatisfied
